# Incentives to yield to Obstetric Referrals in deprived areas of Amansie West district in the Ashanti Region, Ghana

**DOI:** 10.1186/s12939-016-0408-7

**Published:** 2016-07-22

**Authors:** Gladys Buruwaa Nuamah, Peter Agyei-Baffour, Kofi Mensah Akohene, Daniel Boateng, Dominic Dobin, Kwasi Addai-Donkor

**Affiliations:** School of Public Health, Kwame Nkrumah University of Science and Technology, Kumasi, Ghana; Julius Global Health, Julius Center for Health Sciences and Primary Care, University Medical Centre Utrecht, Utrecht, The Netherlands; Amansie West District Health Directorate, Ghana Health Service, Manso Nkwanta, Ghana; Ghana Health Service, Accra, Ghana

**Keywords:** Obstetric referrals, Maternal health, Deprived areas, Inequitable access to healthcare, Amansie West, Ghana

## Abstract

**Background:**

Obstetric referrals, otherwise known as maternal referrals constitute an eminent component of emergency care, and key to ensuring safe delivery and reducing maternal and child mortalities. The efficiency of Obstetric referral systems is however marred by the lack of accessible transportation and socio-economic disparities in access to healthcare. This study evaluated the role of socio-economic factors, perception and transport availability in honouring Obstetric referrals from remote areas to referral centres.

**Methods:**

This was a cross-sectional study, involving 720 confirmed pregnant women randomly sampled from five (5) sub-districts in the Amansie west district in Ghana, from February to May 2015. Data were collected through structured questionnaire using face-to-face interviewing and analyzed using STATA 11.0 for windows. Logistic regression models were fitted to determine the influence of socio-demographic characteristics and pregnancy history on obstetric referrals.

**Results:**

About 21.7 % of the women studied honoured referral by a community health worker to the next level of care. Some of the pregnant women however refused referrals to the next level due to lack of money (58 %) and lack of transport (17 %). A higher household wealth quintile increased the odds of being referred and honouring referral as compared to those in the lowest wealth quintile. Women who perceived their disease conditions as emergencies and severe were also more likely to honour obstetric referrals (OR = 2.3; 95 % CI = 1.3, 3.9).

**Conclusion:**

Clients’ perceptions about severity of health condition and low income remain barriers to seeking healthcare and disincentives to honour obstetric referrals in a setting with inequitable access to healthcare. Implementing social interventions could improve the situation and help attain maternal health targets in deprived areas.

## Background

The progress towards fulfilling the fifth UN Millennium Development Goal (MDG 5), to reduce maternal mortality by three quarters by 2015, is still far off the track in most countries of the world [1]. Despite evidence of effective interventions to promote safe motherhood, morbidity and mortality related to pregnancy and childbirth remain major challenges to stakeholders in health in low-and- middle-income countries. Developed countries have actually succeeded in improving maternal health and reducing maternal mortality. Developing countries on the other hand, are still faced with big inequalities in health within and between different segments of the populations. Disadvantaged groups of women tend to have higher rates of both morbidity and mortality, and less access to safe, affordable and acceptable health care services to enable safe pregnancy and childbirth [1, 2].

Obstetric referral is an important component of emergency care, necessary in reducing maternal mortality. Inability to timely refer expectant mother and refusal of the referred client to honour such referral leads to development of life-threatening complications, often resulting in preventable maternal deaths. Referrals systems connect the lowest level of care to the highest level and ensure access to specialist and continuum of care, as patients are referred from dispensaries and health centers in communities to a secondary level of care [3]. Previous evidence suggests that effective and efficient referral system could reduce stillbirth by 27 %, neonatal deaths by 18 % and maternal deaths by 50 % [4].

In Ghana, the community level represents the largest continuum of the health system and this comprises the community health clinic and the Community-based Health Planning and Services (CHPS), which mainly offer preventive care. This strategy was adopted in 1999 to extend quality Primary Health Care (PHC) to all Ghanaians, especially those in remote areas. It functions in galvanizing local leadership and empowering communities to engage in health outreach activities. A Community Health Officer (CHO) and a nurse are delegated to distant village locations (CHP zones), where they follow clients to their communities and homes to deliver basic health services. The CHO initiates referral to the next level of care during emergencies [5, 6]. The next level of care is the sub-district level, which entails the sub-district management team and health center for managing basic Emergency Obstetric and Neonatal Care (EmONC). From this point, patients are referred to the district referral hospital and then further to the regional hospital.

Maternal health referral systems in Ghana is however fraught with gaps in service proficiency, leading to increased maternal risk. These are exacerbated by socio-economic characteristics, distance to health facility, financial constraints, access to reliable transport services, quality of care and unstructured referral systems [7]. The interplay of these factors influence physician referrals [8–11] and patients’ acceptance to go to the next level [12, 13]. Alter [9], for example, found individuals with high socio-economic status to be more likely to be referred. However, the role of these factors in ensuring smooth referrals in this setting appears to be overlooked.

It is important to note that, the outcome of late obstetric referrals could be detrimental to both the pregnant mother and unborn baby. Pregnancy complications are unpredicted and have the potential to develop rapidly to become severe and life threatening. Previous literature suggests that about two-thirds of women in developing countries deliver at home, without having access to health professionals [14]. Effective referral could prevent the phase II delays (delays in reaching the appropriate facility), as postulated by the three delays model, which describes factors influencing the timely arrival to appropriate care in obstetric emergencies [15]. This study is important, to provide insight into the current obstetric referrals in the Amansie West district. It also adds to existing knowledge on factors that influence obstetric referrals to secondary level of care. This is important to understand which individuals are likely to honour referrals to secondary level of care to help address disparities in access to healthcare.

## Methods

### Study design and setting

A cross-sectional study, which employed quantitative methods, was conducted from February to May 2015 in the Amansie West district in the Ashanti region, Ghana. The district, which has Manso Nkwanta as its capital is one of the 35 districts in the region. It is about 1,364 sq km in size and uniformly rural and deprived. The topography is characterised by waterlogged and dust during the wet and dry seasons respectively. The large expanse of land coupled with rugged relief makes transporting emergencies cases very difficult and sometimes impossible. It takes an average of 5–7 h to transport emergency case from the remote areas to the district hospital at Agroyesum or nearby district hospitals. There is a burden of heavy workload on the few available health staff. The district has a projected population of 149,437 as at 2014 with an annual growth rate of 2.7 %. There are currently 22 health facilities and 54 functional CHPS zones. The doctor to population ratio is 1: 74,719; nurse to population ratio 1:2, 767 and midwife to women in fertility age (WIFA) ratio is 1:4,528. There is a high rate of illiteracy (70 %) and a high dropout rate in schools especially among girls. These conditions are possible risk factors for teenage pregnancies and obstetric complications [16].

### Study population and sampling

The study population consisted of diagnosed second trimester pregnant women presenting at the antenatal clinic who provided written informed consent and satisfying the group-based inclusion criteria.

A multi-stage sampling technique was employed for this study. The antenatal clinics in the study communities were selected from numbers 1 to 10, without replacement by research assistants. At the selected community antenatal clinic, systematic random sampling, guided by a sampling interval (estimated as the required sample size divided by the total attendants), was used to select the required subjects for the study. The sample size was estimated with recourse to Cochran (1977) [17]. Using this formula, and assuming access to referral service at 50 % and a non-response of 20 %, a total of 720 pregnant women were sampled from five towns; one each from the five sub-districts to participate in the study. The required respondents from selected health facilities were proportional to size of total eligible population per community. The distribution of respondents according to the sub district was Manso Nkwanta 120, Edubia 141, Agroyesum114, Antoakrom 140 and Esuowin 205, Table 1.

**Table 1:** Sample distribution from the study communities

Sub-district	Total population	4 % WIRA	Sample size
Antoakrom	17706	708.24	139.95
Agroyesum	14, 428	577.22	114.04
MansoNkwanta	15,187	607.48	120.04
Edubia	17,777	711.08	140.51
Esuonwin	25,997	1039.9	205.48

### Data collection and analysis

A structured questionnaire was used to elicit information on respondents’ socio-demographic and socio-economic characteristics, pregnancy history, experiences and perceptions of obstetric referrals. The questionnaires were pretested among 20 respondents in a sub-district with similar characteristics like the study sub-districts to check for clarity, consistency, and acceptability. The pretested sub-district was excluded from the study to avoid spill over effects. Questionnaires were interviewer administered and the interview results from the field were checked for completeness and internal errors prior to data entry.

Data entry and preliminary analysis were done in EPIDATA and more detailed analysis done with Stata 11.0 for windows [18]. Descriptive statistics were summarized and displayed in graphs and charts. Verified data were cleaned on a regular basis through running programmes on legal values and consistency checks. Data analysis consisted of descriptive statistics, checking of potential assumption violations and testing of the study hypothesis. Continuous variables were compared using student *t*-test and discrete variables analyzed using *χ*^2^ in r X n tables. Logistic regression models were fitted to determine the influence of socio-demographic characteristics and pregnancy history on obstetric referrals, and to control for covariates. The binary outcome (obstetric referral) was based on respondents’ response to the question on whether they honoured referrals for obstetric reasons to the secondary level or not. Two models were fitted; model 1 consisted of only socio-economic variables (education, employment, length of stay in community, household wealth and self-rated socio-economic status) and model 2 involved the addition of pregnancy history (miscarriages and still births) and perception of disease condition. Variables in the multivariable analysis were selected based on *p*-value of less than 0.250 in the univariable analyses. For all analysis, a 2-tailed α with *p* < 0.05 was considered statistically significant.

## Institutional Review Board (IRB)

The protocols of this research were reviewed and cleared by the Committee for Human Resource Publications and Ethics of Kwame Nkrumah University of Science and Technology, Kumasi, Ghana, before the commencement of the study. Prior informed consent was also sought from all participants. All recruited patients and health personnel signed an informed consent and permission was sought from the District Health Directorate, Amansie West and the opinion leaders of study communities.

## Results

### Socio-demographic characteristics and pregnancy histories

Table 2 shows the results of background characteristics of respondents in this study. The median age of the women in this study was 25 years and majority, 57.5 % were in the age range of 20 to 29 years. Most women had basic education (primary, JHS or middle school). About 17 % had no formal education and only 1.4 % had tertiary education. With respect to their marital status, 65.5 % were cohabitating whereas 28.8 % were married. More than three-fourth of the women were employed and 25.1 and 28.3 % of them were farmers and traders respectively. Two hundred respondents, constituting 27.8 % had no child whereas 19.6 % had more than three children. About 97 % had registered with the NHIS and almost all of them with the exception of two were active members of the scheme. The mean length of stay in the community was 15.5 years (SD = 11.0) and 34.9 % had lived in the community for more than 20 years. The distribution of wealth, by quintiles was quite proportional among the women, with 21.4 and 27.3 % distributed in the lowest and highest quintiles respectively. Most of the women rated themselves as moderately rich whereas 6.3 % rated themselves as very poor. About 57.2 % disclosed that they spoke to their husbands before seeking healthcare or honouring a referral.

**Table 2:** Background characteristics and univariable associations with obstetric referrals

Variables	Frequency *N* = 720	Percentage	Referral to secondary level		*p*-value
			Yes, %	No, %	
Age					
<20	99	13.8	19.2	80.8	0.723
20–29	414	57.5	22.2	77.8	
30–40	191	26.5	22.6	77.4	
>40	16	2.2	12.5	87.5	
Highest educational level					
None	121	16.8	22.5	77.5	0.011
Primary	148	20.6	31.1	68.9	
Middle/JHS	357	49.6	19.3	80.7	
Secondary/SHS	84	11.7	16.7	83.3	
Tertiary/others	10	1.4	0.0	100.0	
Marital status					
Married	208	28.9	20.7	79.3	0.912
Cohabitation	457	63.5	22.1	77.9	
Single/Divorced	55	7.6	21.8	78.2	
Occupational status					
Trading	180	25.1	26.6	73.4	0.068
Farming	203	28.3	23.2	76.8	
Public service/ Small Scale mining/Galamsey/banker/other	176	24.5	15.6	84.4	
Unemployed/student	158	22.0	20.4	79.6	
Registered with NHIS					
Yes	697	97.1	22.0	78.0	0.530
No	23	2.9	14.3	85.7	
Length of stay in community					
<5	188	26.1	17.0	83.0	0.187
5–10	112	15.6	27.7	72.3	
10–15	43	6.0	16.3	83.7	
15–20	126	17.5	24.6	75.4	
>20	251	34.9	22.0	78.0	
Household wealth					
First quintile (lowest)	137	21.4	16.1	83.9	0.085
Second quintile	187	29.2	23.5	76.5	
Third quintile	142	22.1	19.7	80.3	
Fourth quintile (Highest)	175	27.3	27.6	72.4	
Self-rated socio-economic status					
Rich	46	6.4	34.8	65.2	0.002
Moderately rich	486	67.5	17.7	82.3	
Poor	143	19.9	27.3	72.7	
Very poor	45	6.3	33.3	66.7	

Test: Chi-square/Fischer’s exact test

The pregnancy histories of the women are presented in Table 3. The gestational age of majority of the women was four to eight weeks and about 65 % had had three pregnancies or more. About 29.2 % of 537 respondents had had no live births whereas 19.2 % had three or more live births. Most of the women, 77.4 %, had never experienced miscarriages and 90.9 % had no stillbirths. About 99.2 and 74.6 % of respondents had no ectopic pregnancies and induced abortions respectively. Oedema was the most cited disease condition experienced by the women during pregnancy (23 %), followed by cough (18.6 %) and most of the respondents, 79.8 %, disclosed that they did not perceive their disease conditions as emergencies.

**Table 3:** Pregnancy histories of study population and univariable associations with obstetric referrals

Variables	Frequency *N* = 720	Percentage	Referred to next level of healthcare		*p*-value
			Yes, %	No, %	
Total pregnancies (*n* = 526)					
− 1	86	16.3	20.0	80.0	0.369
− 2	98	18.6	20.4	79.6	
− 3	106	20.2	28.3	71.7	
− >3	236	44.9	26.7	73.3	
Live births (*n* = 527)					
− 0	154	29.2	22.9	77.1	0.502
− 1	120	22.8	22.5	77.5	
− 2	80	15.2	25.0	75.0	
− 3	72	13.7	22.2	77.8	
− >3	101	19.2	31.7	68.3	
Miscarriages (*n* = 526)					
− None	407	77.4	23.2	76.8	0.081
− 1	90	17.1	26.7	73.3	
− 2 or more	29	5.5	41.4	58.6	
Still births (*n* = 525)					
− None	477	90.9	23.9	76.1	0.211
− 1	32	6.1	37.5	62.5	
− 2 or more	16	3.0	33.3	66.7	
Induced abortions (*n* = 523)					
− None	390	74.6	26.0	74,0	0.590
− 1	100	19.1	23.0	77.0	
− 2 or more	3	6.3	18.2	81.8	
Disease conditions during pregnancy (*n* = 522)					
− Oedema	120	23.0	18.5	81.5	0.257
− Cough	97	18.6	24.7	75.3	
− Rashes	35	6.7	25.7	74.3	
− Malaria	97	18.0	23.5	76.6	
− Other	176	33.7	30.1	69.9	
Perception about disease conditions (*n* = 554)					
− Emergencies	112	20.2	33.9	66.1	0.005
− Not emergencies	443	79.8	21.3	78.7	

Test: Chi-square/Fischer’s exact test

### Differences in background characteristics and pregnancy history between outcome groups

Tables 2 and 3 also summarise the comparison between women who were referred or not with respect to their socio-demographic characteristics and pregnancy history. As shown in Table 2, maternal health referral was associated with educational level (*p* = 0.011) and self-rated socio-economic status (*p* = 0.002). Obstetric referral was shown to decrease with increasing level of education. The age of the mothers, marital status, occupation, NHIS subscription, duration of stay in community, and health wealth quintile had no influence on maternal referrals in the district. Obstetric referrals to secondary level, was influenced by women’s perception of their disease conditions. Women, who perceived their disease conditions to be emergencies or severe were more likely to honour their referral to the next level (33.9 % versus 21.3 %; *p* = 0.005). The number of stillbirths, induced abortions, miscarriages and live births were not significantly different between the two outcome groups.

### Obstetric referrals and access to transportation during referrals

**Fig. 1:**
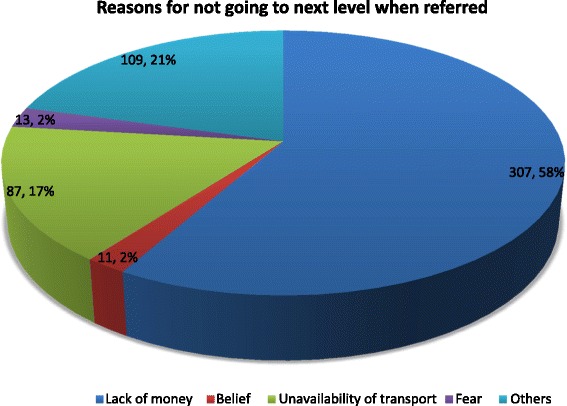
Disincentives to honour referrals to the next level of healthcare

Table 4 presents results on obstetric referrals in the Amansie West district, Ashanti region. It also shows results on access to transportation services during emergencies. Most of them disclosed that they were seen or referred for conditions other than malaria or vomiting. More than 90 % disclosed that they always meet staff at the referral point and about 76.6 % indicated that the health staffs at the referral points were able to solve the problems. About 22 % of the women had ever been referred to the next level of care. Most of the women who are referred to health center or maternity home were not further referred to the next level whereas 3.2 % were always referred. The womens’ reasons for not going to the next level when referred included lack of money (58 %), lack of transport (17 %) and other reasons (21 %) as shown in Fig. 1. Majority, 61.9 % of the women felt referrals should be only for severe conditions or in situations when logistics are inadequate (34.7 %). Among 637 respondents, 95.8 % disclosed that transport services were available whenever needed for emergencies and commercial cars was the most cited emergency transport service available (88.2 %) whereas only 6.6 % cited ambulance. Only 26.8 % of the women had access to an ambulance during emergencies and about 15 % spent up to an hour to arrange for transport during emergencies. The health facility was quite proximal to more than half of the women, who spent less than 30 min to reach the health facility.

**Table 4:** Maternal health referrals

Variables	Frequency	Percentage
Ever seen by community health extension worker		
− Yes	72	10.0
Meeting health staff at the referred point (Maternity, CHPS and Heath Center, chemical seller) (*n* = 543)		
− All the time	505	93.0
− Most of the time	28	5.6
− Some of the time	9	1.7
− Rarely	1	0.2
Health staffs ability to solve problems (*n* = 350)		
− All the time	268	76.6
− Most of the time	63	18.0
− Some of the time	19	5.4
Ever referred by community health extension workers	156	21.7
Personal feeling about referrals (*n* = 525)		
− Severe condition	325	61.9
− Inadequate logistics/equipment	182	34.7
− Fear	15	2.9
− Other	3	0.6
Availability of transport services for emergencies (*n* = 637)		
− Yes	610	95.8
Means of transport when referred to next level (*n* = 707)		
− Commercial cars	624	88.2
− Ambulance	48	6.8
− Motor bikes	29	4.1
− Others	6	0.8
Access to ambulance services when needed for emergencies (*n* = 717)	192	26.8
Length of time to have transportation when referred (in minutes) (*n* = 708)		
− <30	586	82.8
− 30 – 60	106	15.0
− >60	16	2.3
*Mean (SD)*	*13.4 (9.2)*	
*Range*	*0–120*	
Total cost of medical care at the clinic in last nine month (*n* = 718)		
− *Min/max*	0.00/1001.00	
− *Mean (SD)*	19.50 (47.1)

### Predictors of obstetric referrals

In Table 5, we show the results of multivariable regression analysis of the influence of socio-demographic characteristics and pregnancy history on the odds of honouring referral to the secondary level of health care. In model 1, where the socio-demographic variables were controlled for each other, household wealth quintile and self-rating of socio-economic status had significant influence on maternal health referrals. Mothers who were in the second and fourth quintiles were more likely to be referred and accordingly honour the referrals as compared to those in the lowest quintile. These odds ratios increased in model 2 when pregnancy histories were introduced into the model. Women who were in the second quintile were 4.7 times more likely to be referred and yielded to referral advice as compared to those in the lowest quintile (OR = 4.7; 95 % CI = 2.0–10.8). In model 2, women who perceived their disease conditions as emergencies or severe were 2.3 times more likely to yield to referrals to the secondary level as compared to those who did not (OR = 2.3; 95 % CI = 1.3–3.9).

**Table 5:** Multivariable logistic regression analyses of factors influencing maternal health referrals

*Variables*	Model 1 AOR [95 % CI]	Model 2 AOR [95 % CI]
*A. Socio-economic*		
Highest educational level (ref = none)		
− Primary	1.7 [0.9, 3.2]	1.4 [0.7, 2.9]
− Middle/JHS	0.8 [0.4, 1.3]	0.8 [0.4, 1.6]
− Secondary/Tertiary/others	0.6 [0.3, 1.3]	0.6 [0.2, 1.5]
Employed	1.1 [0.6, 1.9]	1.2 [0.6, 2.4]
Length of stay in community	1.0 [1.0, 1.0]	1.0 [1.0, 1.0]
Household wealth (ref = first)		
− Second quintile	2.7 [1.4, 5.1]**	4.7 [2.0, 10.8]***
− Third quintile	1.6 [0.8, 3.1]	2.9 [1.2, 6.8]*
− Fourth quintile (Highest)	2.2 [1.2, 4.3]*	2.9 [1.2, 6.6]*
Self-rated socio-economic status (ref = Rich)		
− Moderately rich	0.4 [0.2, 0.9]*	0.4 [0.2, 1.0]
− Poor/very poor	1.0 [0.4, 2.2]	0.8 [0.3, 2.3]
*B. Pregnancy history and perception of disease*		
One or more miscarriages		1.3 [0.8, 2.2]
One or more still births		1.1 [0.5, 2.4]
Perceive disease conditions as emergencies		2.3 [1.3, 3.9]**

Outcome: maternal health referral; *p < 0.05; **p < 0.01; ***p < 0.001

## Discussion

Efficient referral systems are very essential in maternal healthcare to ensure a smooth linkage between the lower levels of care, which provides mainly preventive services with the higher specialized healthcare delivery. This study sought to gain insight into the referral systems at the Amansie West district in Ghana, and to assess the influence of socio-economic factors, transport availability and pregnancy history on obstetric referrals. This study had two main findings. First, the study found transportation as an important factor in effective referral system. Access to ambulance was quite low and difficulty in accessing transportation barred some pregnant women from accepting and honouring referrals. Second, socio-economic status significantly influenced community health worker’s decision of who should be referred for secondary care and to a large extent who honours referral. Respondents in the higher wealth quintile were more likely to be referred and honoured as compared to those in the lowest quintile. Obstetric referrals were found to be low in the Amansie West district, with only 21.7 % of the women interviewed ever being referred to the next level of care.

Patients’ perception of their disease conditions influenced their willingness to accept referral to the next level of care in this study. The most cited disease condition was oedema followed by cough and malaria. Malaria was however the leading cause of OPD attendance in the district in 2013 [16]. In a similar vein, most of the women indicated that they were referred to the next level of care for conditions other than malaria. The district has only one (1) district hospital and six (6) health centres. The health centres are however not well equipped and the chances are that they may make unnecessary referrals.

Transportation is an important factor in healthcare delivery, especially in cases of emergency. Rural and remote regions in developing countries have limited access to regular and suitable transport, making physical access to specialized healthcare difficult or impossible in some cases. Many developing countries’ studies, identified transport cost, availability of transport and travel time as strong correlates of health care utilization [19, 20]. Rural dwellers often have greater distances to cover in order to reach health care services [21] and transport costs are relatively higher [19]. Under the current CHPS strategy in Ghana, transportation is paramount since the services provided at the CHPS zones are basically preventive and there is the need to ensure reliable means for referrals.

In this study, access to reliable transportation appeared to be an issue for most of the women. Although majority indicated that transport services were available, most of these were commercial vehicles and only a few had access to ambulances during emergencies. Arranging for transport could take about 30 min to an hour for some of the respondents. This finding is parallel with previous evidence where participants narrated their difficulties in having access to transportation and in some instances, were forced to deliver at home [22, 23]. This has been cited as a drawback of the CHPS strategy; failing to take into consideration all the important aspects of health delivery and most importantly making provision for effective referrals. For this reason, some patients are forced to refuse referrals to the next level of care, which is very characteristic of a health care with weak ambulatory system. The Amansie west district has very poor road network in most parts of the district such that when distances to facilities are even short, vehicles would not like to ply those roads.

The role of socio-economic disparities in health care in limited resource settings has long been confirmed through research. Investigating into the socio-economic differences in different aspects of health delivery is important to ensure equitable distribution of healthcare to all people. This study found that wealth status could have influence on who is being referred to the next level of care and whether the referred patient will honour such referrals. The distribution of respondents according to wealth quintiles was quite uniform in this study and majority of the women rated themselves as moderately rich. As compared to women in the lowest wealth quintile, belonging to the second and fourth wealth quintiles significantly increased the likelihood of being referred to the next level of care.

As suggested in previous studies, this could be explained that women with high socio-economic status tend to be more knowledgeable about their illness, have greater expectations of the level of care and could better negotiate with their primary care physician to maximize their referral and access the next level of care [9]. From another viewpoint,, community healthcare workers refer mothers they believe have the financial resources to make it to the secondary level, putting those in the lowest wealth quintile at a disadvantage. Studies from other settings have also reported physicians’ lack of motivation to refer patients who are not on medical insurance, with the belief that they could not afford cost of care at the next level [10, 11]. This is however, discriminatory and should be discouraged by addressing the structural problems of road network and ambulatory system. It is expensive in terms of transportation and cost of healthcare to be referred to the next level and those in the high wealth quintiles honoured obstetric referrals because they had the means to [9]. As put forward by Buor [19], the use of health services depends on one’s ability to pay. Low incomes had been captured as a barrier to seeking healthcare and can create a devastating financial burden for some people [24], especially in a setting with inequitable access to healthcare. A lack of finance could therefore have adverse effect on health care seeking [25, 26]. That is although the willingness to pay for services might exist [27], the means to do so, may not. On the other hand, Chan [8], found physician referrals to be higher among low socio-economic status individuals and this they explained that this group of patients were likely to have more illness.

The age, educational, employment, length of stay in community being an active member of NHIS appeared not to be important in health workers decision to refer patients to the next level. The implications for these in the attainment of maternal health targets could be devastating. Probably implementing pro-distance and referral intervention health care delivery strategy could be helpful.

## Limitations of the study

The choice of cross-sectional study design could not permit us to make direct inferences on the relationship between maternal referrals and the covariates studied. This study might also have suffered some recall bias in responses from participants. We however believed that this was not much and could not affect the findings and conclusions of the study, since most of the questions pertained to their recently past pregnancies.

## Conclusions

Incentives to yield to obstetric referrals to the next level in deprived areas were found to depend on health staff’s decision as to who to refer, clients’ perception about severity of health condition, availability of transportation and high-income level of the client. The absence of these are disincentives to honour obstetric referrals to the next level in a setting with inequitable access to healthcare. Implementing social interventions and improving ambulatory system could improve the situation and help attain maternal health targets in deprived areas. The health centres are faced with logistical constraints and are unable to manage primary health conditions. The CHPS compounds, CHCs, community clinics and maternity homes must therefore be well equipped to offer the requisite primary health care for pregnant women and refer to the district hospital only when appropriate.

## References

[1] Nations U (2011). The Millennium Development Goals Report 2011.

[2] Irwin A, Valentine N, Brown C, Loewenson R, Solar O, Brown H, Koller T, Vega J (2006). The commission on social determinants of health: tackling the social roots of health inequities. PLoS Med.

[3] Macintyre K, Hotchkiss DR (1999). Referral revisited: community financing schemes and emergency transport in rural Africa. SocSciMed.

[4] Pattinson R, Kerber K, Buchmann E, Friberg IK, Belizan M, Lansky S (2011). Stillbirths: How can health systems deliver for mothers and babies?. Lancet.

[5] Awoonor-Williams JK, Bawah AA, Nyonator FK, Asuru R, Oduro A, Ofosu A (2013). The Ghana essential health interventions program: a plausibility trial of the impact of health systems strengthening on maternal & child survival. BMC Health Serv Res.

[6] Dil Y, Strachan D, Cairncross S, Korkor AS, Hill Z (2012). Motivations and challenges of community-based surveillance volunteers in the northern region of Ghana. J Community Health.

[7] Awoonor-Williams JK (2010). Transportation and Referral for Maternal Health Within the CHPS System in Ghana.

[8] Chan BTB, Austin PC (2003). Patient, physician and community factors affecting referrals to specialist in Ontaria, Canada; a population-based, multi-level modelling approach. Med Care.

[9] Alter DA, Naylor CD, Austin P (1999). Effects of socioeconomic status on access to invasive cardiac procedures and on mortality after acute myocardial infarction. N Engl J Med.

[10] Forrest CB, Nutting PA, von Schrader S, Rohde C, Starfield B (2006). Primary care physician specialty referral decision making: patient, physician, and health care system determinants. Med Decis Making.

[11] Institute of Medicine (2002). Care without Coverage: Too Little, Too Late.

[12] Hanson C, Cox J, Mbaruku G, Manzi F, Gabrysch S, Schellenberg D (2015). Maternal mortality and distance to facility-based obstetric care in rural southern Tanzania: a secondary analysis of cross-sectional census data in 226 000 households. Lancet Glob Health.

[13] Gabrysch S, Campbell OMR (2009). Still too far to walk: literature review of the determinants of delivery service use. BMC Pregnancy Childbirth.

[14] The United Nations Children’s Fund (2009). State of the world’s Children.

[15] Thaddeus S, Maine D (1994). Too far to walk: maternal mortality in context. Soc Sc Med.

[16] Amansie West District (2015). District Profile, 2014.

[17] Cochran WG (1997). Sampling Techniques.

[18] StataCorp (2009). Stata Statistical Software: Release 11.

[19] Buor D (2003). Analysing the primacy of distance in the utilization of health services in the Ahafo-Ano South district, Ghana. Int J Health Plann Manage.

[20] Witter S, Osiga G (2004). Health service quality and users’ perceptions in West Nile, Uganda. Int J Health Plann Manage.

[21] Noor AM, Zurovac D, Hay SI, Ochola SA, Snow RW (2003). Defining equity in physical access to clinical services using geographical information systems as part of malaria planning and monitoring in Kenya. Tropical Med Int Health.

[22] Atuoye KN, Dixon J, Rishworth A, Galaa SZ, Boamah SA, Luginaah I (2015). Can she make it? Transportation barriers to accessing maternal and child health care services in rural Ghana. BMC Health Serv Res.

[23] Afari H, Hirschhorn LR, Michaelis A, Barker P, Sodzi-Tettey S. Quality improvement in emergency obstetric referrals: qualitative study of provider perspectives in Assin North district, Ghana. BMJ Open. 2014;4. 10.1136/bmjopen-2014-005052 PMC4025473 24833695

[24] Gotsadze G, Bennet S, Ranson K, Gzirishvili D (2005). Health care-seeking behaviour and out-of-pocket payments in Tbilisi, Georgia. Health Policy Plan.

[25] Taffa N, Chepngeno G (2005). Determinants of health care seeking for childhood illnesses in Nairobi slums. Tropical Med Int Health.

[26] Nwameme AU, Philips JF, Adongo PB (2014). Compliance with emergency obstetric care referrals among pregnant women in an urban informal settlement of Accra, Ghana. Matern Child Health J.

[27] Foreit JR, Foreit KJF (2003). The reliability and validity of willingness to pay surveys for reproductive health pricing decisions in developing countries. Health Policy.

